# Supplementary material for: Association of SARS-CoV-2 BA.4/BA.5 Omicron lineages with immune escape and clinical outcome
Source: Nat Commun. 2023 Mar 14;14:1407. doi: 10.1038/s41467-023-37051-5 (PMC10012300; doi:10.1038/s41467-023-37051-5)
Supplement: Supplementary file 1 — Supplementary Information [file 41467_2023_37051_MOESM1_ESM.pdf]

Table S1: Diagnosis codes used to identify acute respiratory infection-associated hospital admissions.

Code	Diagnosis
A48.1	Legionnaire's disease
B34.2	Coronavirus infection (unspecified)
B44.0	Invasive pulmonary aspergillosis
B97.29	Other coronavirus as the cause of diseases classified elsewhere
J00	Acute nasopharyngitis (common cold)
J01.00	Acute maxillary sinusitis, unspecified
J01.10	Acute frontal sinusitis, unspecified
J01.20	Acute ethmoidal sinusitis, unspecified
J01.30	Acute sphenoidal sinusitis, unspecified
J01.40	Acute pansinusitis, unspecified
J01.80	Other acute sinusitis
J01.90	Acute sinusitis, unspecified
J02.0	Streptococcal pharyngitis
J02.8	Acute pharyngitis due to other specified organisms
J02.9	Acute pharyngitis, unspecified
J03.00	Acute streptococcal tonsillitis, unspecified
J03.90	Acute tonsillitis, unspecified
J04.0	Acute laryngitis
J04.10	Acute tracheitis without obstruction
J05.0	Acute obstructive laryngitis (croup)
J05.10	Acute epiglottitis without obstruction
J06.0	Acute laryngopharyngitis
J06.9	Acute upper respiratory infection, unspecified
J09.X1	Influenza due to identified novel influenza A virus with pneumonia
J09.X2	Influenza due to identified novel influenza A virus with other respiratory manifestations
J10.00	Influenza due to other identified influenza virus with unspecified type of pneumonia
J10.01	Influenza due to other identified influenza virus with same other identified influenza virus pneumonia
J10.08	Influenza due to other identified influenza virus with other pneumonia
J10.1	Influenza due to other identified influenza virus with other respiratory manifestations
J10.2	Influenza due to other identified influenza virus with gastrointestinal manifestations
J11.00	Influenza due to unidentified influenza virus with unspecified type of pneumonia
J11.08	Influenza due to unidentified influenza virus with specified pneumonia
J11.1	Influenza due to unidentified influenza virus with other respiratory manifestations
J12.1	Respiratory syncytial virus pneumonia
J12.2	Parainfluenza virus pneumonia
J12.3	Human metapneumovirus pneumonia
J12.81	Pneumonia due to SARS-associated coronavirus
J12.82	Pneumonia due to coronavirus disease 2019
J12.89	Other viral pneumonia
J12.9	Viral pneumonia, unspecified
J13	Pneumonia due to *Streptococcus pneumoniae*
J14	Pneumonia due to *Haemophilus influenzae*
J15.0	Pneumonia due to *Klebsiella pneumoniae*
J15.1	Pneumonia due to *Pseudomonas*
J15.20	Pneumonia due to *Staphylococcus*, unspecified
J15.211	Pneumonia due to methicillin susceptible *Staphylococcus aureus*
J15.212	Pneumonia due to methicillin resistant *Staphylococcus aureus*
J15.4	Pneumonia due to other *Streptococci*
J15.5	Pneumonia due to *Escherichia coli*
J15.6	Pneumonia due to other aerobic gram-negative bacteria
J15.7	Pneumonia due to *Mycoplasma pneumoniae*
J15.8	Pneumonia due to other specified bacteria
J15.9	Unspecified bacterial pneumonia
J16.8	Pneumonia due to other specified infectious organisms
J18.0	Bronchopneumonia, unspecified organism
J18.1	Lobar pneumonia, unspecified organism
J18.8	Other pneumonia, unspecified organism
J18.9	Pneumonia, unspecified organism
J20.2	Acute bronchitis due to *Streptococcus*
J20.5	Acute bronchitis due to respiratory syncytial virus
J20.6	Acute bronchitis due to rhinovirus
J20.8	Acute bronchitis due to other specified organisms
J20.9	Acute bronchitis, unspecified
J22	Unspecified acute lower respiratory infection
J39.0	Retropharyngeal and parapharyngeal abscess
J39.1	Other abscess of pharynx
J39.2	Other diseases of pharynx
J39.8	Other specified diseases of upper respiratory tract
J80	Acute respiratory distress syndrome
J96.00	Acute respiratory failure, unspecified with hypoxia or hypercapnia
J96.01	Acute respiratory failure with hypoxia
J96.02	Acute respiratory failure with hypercapnia
J96.10	Chronic respiratory failure, unspecified with hypoxia or hypercapnia
J96.11	Chronic respiratory failure with hypoxia
J96.12	Chronic respiratory failure with hypercapnia

J96.20	Acute and chronic respiratory failure, unspecified with hypoxia or hypercapnia
J96.21	Acute and chronic respiratory failure with hypoxia
J96.22	Acute and chronic respiratory failure with hypercapnia
J96.90	Respiratory failure, unspecified with hypoxia or hypercapnia
J96.91	Respiratory failure with hypoxia
J96.92	Respiratory failure with hypercapnia
M35.81	Multisystem inflammatory syndrome
M35.89	Other specified systemic involvement of connective tissue
R05.1	Acute cough
R05.3	Chronic cough
R05.8	Other specified cough
R05.9	Cough, unspecified
R09.2	Respiratory arrest
R50.9	Fever, unspecified
U07.1	COVID-19

Table S2: Predictive validity of S gene target failure for lineage classification, among sequenced specimens.

Month	S gene detection	Lineage (identified by sequencing), %					Predictive validity of SGTF, *n*/*N* (%)
		BA.1	BA.2	BA.4	BA.5	Other	
May, 2022							
	S gene detected	0 (0.0)	620 (95.5)	1 (0.9)	0 (0.0)	3 (100.0)	620/624 (99.4)
	S gene target failure (SGTF)	7 (100.0)	29 (4.5)	112 (99.1)	114 (100.0)	0	226/262 (86.3)
June, 2022							
	S gene detected	0	600 (97.2)	1 (0.6)	6 (1.4)	2 (100.0)	600/609 (98.5)
	S gene target failure (SGTF)	0	17 (2.8)	158 (99.4)	425 (98.6)	0 (0.0)	583/600 (97.2)
July, 2022							
	S gene detected	0	375 (99.2)	0 (0.0)	12 (3.6)	0	375/387 (96.9)
	S gene target failure (SGTF)	0	3 (0.8)	64 (100.0)	323 (96.4)	0	387/390 (99.2)
May through July, 2022							
	S gene detected	0 (0.0)	1,595 (97.0)	2 (0.6)	18 (2.0)	5 (100.0)	1,595/1,620 (98.5)
	S gene target failure (SGTF)	7 (100.0)	49 (3.0)	334 (99.4)	862 (98.0)	0 (0.0)	1,196/1,252 (95.5)

SGTF: S gene target failure, defined as a cycle threshold value ≥37 for the S gene probe in samples with cycle threshold <37 for the N and ORF1ab gene probes. Specimens were selected at random for sequencing.

Table S3: Likelihood of sequencing failure among specimens with and without S gene target failure.

**Month**	**S gene detection**	Specimens submitted, *N*	**Sequencing result** Pass, *n* (%)	Fail, *n* (%)
May, 2022				
	S gene detected	1,110	623 (56.1)	487 (43.9)
	S gene target failure (SGTF)	611	262 (42.9)	349 (57.1)
June, 2022				
	S gene detected	1,049	609 (58.1)	440 (41.9)
	S gene target failure (SGTF)	1,052	599 (56.9)	453 (43.0)
July, 2022				
	S gene detected	671	387 (57.7)	284 (42.3)
	S gene target failure (SGTF)	672	389 (57.9)	283 (42.1)
May through July, 2022				
	S gene detected	2,830	1619 (57.2)	1211 (42.8)
	S gene target failure (SGTF)	2,335	1,250 (53.5)	1,085 (46.4)

SGTF: S gene target failure, defined as a cycle threshold value ≥37 for the S gene probe in samples with cycle threshold <37 for the N and ORF1ab gene probes. Specimens were selected at random for sequencing.

Table S4: Prior vaccination, documented SARS-CoV-2 infection, and hybrid immunity among cases with BA.2 and BA.4/BA.5 lineage SARS-CoV-2 infection.

**Characteristic**	***n* (%)**		**OR (95% CI)**	
	BA.2 (No SGTF)	BA.4/BA.5 (SGTF)	Unadjusted OR[3]	Adjusted OR[3,4]
	N=46,976	*N*=59,556		
0 doses, no documented prior infection	6,917 (14.7)	8,168 (13.7)	ref.	ref.
0 doses, documented prior infection	626 (1.3)	1,254 (2.1)	1.52 (1.40, 1.65)	1.55 (1.43, 1.69)
1 doses, no documented prior infection	1,076 (2.3)	1,363 (2.3)	1.09 (0.97, 1.21)	1.09 (0.98, 1.23)
1 doses, documented prior infection	45 (0.1)	140 (0.2)	1.65 (1.43, 1.89)	1.70 (1.47, 1.96)
2 doses, no documented prior infection	11,340 (24.1)	13,219 (22.2)	1.02 (0.97, 1.07)	1.04 (0.98, 1.10)
2 doses, documented prior infection	403 (0.9)	819 (1.4)	1.55 (1.40, 1.71)	1.61 (1.45, 1.80)
3 doses, no documented prior infection	23,122 (49.2)	28,050 (47.1)	1.08 (1.03, 1.13)	1.15 (1.09, 1.21)
3 doses, documented prior infection	355 (0.8)	852 (1.4)	1.64 (1.49, 1.81)	1.78 (1.60, 1.98)
≥4 doses, no documented prior infection	3,063 (6.5)	5,617 (9.4)	1.14 (1.06, 1.22)	1.38 (1.27, 1.49)
≥4 doses, documented prior infection	29 (0.1)	74 (0.1)	1.73 (1.55, 1.92)	2.14 (1.89, 2.42)

SGTF: S gene target failure, here interpreted as a proxy for SARS-CoV-2 lineage; CI: Confidence interval; OR: Odds ratio.

[1]Vaccine doses received are summed across all products.

[2]Cases were excluded in the event of any positive test result in the previous 90 days. Sensitivity analyses subset to cases with documented prior infection, and exploring bias resulting from potential misclassification of prior infection status, are presented in **Table S7** and **Figure S1**.

[3]Odds ratios and adjusted odds ratios are estimated using logistic regression models defining cases' calendar week (or weekend) of diagnosis as strata. Adjusted estimates control for all variables listed in **Table 1** as covariates with the exception of Paxlovid receipt.

[4]We did not identify statistically-significant evidence of interaction between prior documented infection and vaccination; estimates of the interaction parameters, describing the relative increase in adjusted odds of prior documented infection among BA.4/BA.5 (vs. BA.2) cases among recipients of 1, 2, 3, or ≥4 doses, as compared to recipients of 0 doses, were 1.53 (0.95-2.48), 0.90 (0.73-1.11), 0.95 (0.77-1.18), and 0.61 (0.35-1.07), respectively.

Table S5: Association of infecting lineage with risk of severe clinical outcomes over 15 days after diagnosis among cases tested 29 April, 2022 to 29 July, 2022.

Clinical endpoint	Infecting lineage	Events		Hazard ratio (95% CI)	
		n (%)[1]	Rate per 100,000 person-days	Unadjusted	Adjusted
Emergency department presentation—15 days					
	BA.2 (*S gene detected*)	859 (1.7)	128.0	ref.	ref.
	BA.4/BA.5 (*S gene target failure*)	1,013 (1.8)	119.5	0.90 (0.79, 1.01)	0.95 (0.84, 1.07)
Hospital admission—15 days					
	BA.2 (*S gene detected*)	94 (0.20)	13.3	ref.	ref.
	BA.4/BA.5 (*S gene target failure*)	121 (0.20)	13.9	0.92 (0.64, 1.32)	0.96 (0.73, 1.27)

CI: Confidence interval. Estimates indicate the adjusted hazard ratios (aHR) of each outcome, comparing cases with BA.4/BA.5 infection to those with BA.2 infection, estimated via Cox proportional hazards models including strata for cases' week of diagnosis and all covariates listed in **Table 1** and **Table 2**.

[1]Proportions calculated among 46,976 BA.2 cases and 59,556 BA.4/BA.5 cases followed ≥30 days (for endpoints of emergency department presentation and hospital admission).

Table S6: Unadjusted association of prior vaccination or infection with risk of severe clinical outcomes among cases tested 29 April, 2022 to 29 July, 2022.

Population	Characteristic	Unadjusted hazard ratio (95% CI), by clinical endpoint[1]			
		All-cause ED presentation (30 days)	*All-cause hospital admission (30 days)*	*ARI-associated hospital admission (30 days)*	*ICU admission (60 days)*
All cases					
	0 vaccine doses	ref.	ref.	ref.	ref.
	2 vaccine doses	0.76 (0.64, 0.90)	0.79 (0.48, 1.30)	0.73 (0.42, 1.27)	1.33 (0.24, 7.24)
	3 vaccine doses	0.83 (0.71, 0.96)	0.73 (0.47, 1.13)	0.76 (0.47, 1.24)	1.72 (0.38, 7.87)
	≥4 vaccine doses	1.21 (0.97, 1.54)	1.17 (0.58, 2.34)	1.40 (0.69, 2.86)	1.67 (0.15, 18.5)
	No documented prior infection	ref.	ref.	ref.	ref.
	Any documented prior infection	1.08 (0.79, 1.47)	2.12 (1.08, 4.18)	2.21 (1.08, 4.53)	2.06 (0.23, 15.5)
BA.4/BA.5 cases					
	0 vaccine doses	ref.	ref.	ref.	– –
	2 vaccine doses	0.83 (0.56, 1.21)	1.10 (0.40, 3.05)	0.93 (0.30, 2.93)	– –
	3 vaccine doses	0.83 (0.59, 1.15)	0.50 (0.18, 1.41)	0.60 (0.20, 1.78)	– –
	≥4 vaccine doses	1.01 (0.60, 1.71)	0.40 (0.05, 3.34)	0.47 (0.05, 4.05)	– –
	No documented prior infection	ref.	ref.	ref.	– –
	Any documented prior infection	0.78 (0.40, 1.52)	1.76 (0.42, 7.47)	2.12 (0.49, 9.11)	– –
BA.2 cases					
	0 vaccine doses	ref.	ref.	ref.	ref.
	2 vaccine doses	0.75 (0.62, 0.90)	0.70 (0.40, 1.24)	0.67 (0.36, 1.27)	1.00 (0.17, 5.97)
	3 vaccine doses	0.83 (0.70, 0.98)	0.78 (0.48, 1.28)	0.80 (0.47, 1.38)	1.56 (0.34, 7.20)
	≥4 vaccine doses	1.29 (0.99, 1.67)	1.41 (0.67, 2.97)	1.70 (0.79, 3.67)	1.78 (0.16, 19.6)
	No documented prior infection	ref.	ref.	ref.	ref.
	Any documented prior infection	1.19 (0.84, 1.69)	2.25 (1.05, 4.84)	2.26 (0.99, 5.15)	2.51 (0.33, 19.1)

CI: Confidence interval. Estimates indicate the unadjusted hazard ratios (aHR) of each outcome, comparing cases with BA.4/BA.5 infection to those with BA.2 infection, estimated via Cox proportional hazards models including strata for cases' week of diagnosis without additional covariates.

[2]Previous infection defined by any positive test result or diagnosis ≥90 days prior to the date of the current test. We omit estimates among recipients of single vaccine doses due to sparse sample sizes (*N*=1,121 BA.4/BA.5 cases and 1,503 BA.2 cases).

Table S7: Association of prior vaccination or infection with risk of severe clinical outcomes over 15 days after diagnosis among cases tested 29 April, 2022 to 29 July, 2022.

Population	Characteristic	Adjusted hazard ratio (95% CI), by clinical endpoint[1]	
		All-cause ED presentation—15 days 15 days	*All-cause hospital admission—30 days* 15 days
All cases			
	0 vaccine doses	ref.	ref.
	2 vaccine doses	0.79 (0.68, 0.90)	0.82 (0.69, 1.15)
	3 vaccine doses	0.67 (0.63, 0.70)	0.58 (0.41, 0.66)
	4 vaccine doses	0.58 (0.53, 0.72)	0.43 (0.27, 0.63)
	No documented prior infection	ref.	ref.
	Any documented prior infection	0.74 (0.65, 0.84)	0.77 (0.54, 1.20)
BA.4/BA.5 cases			
	0 vaccine doses	ref.	ref.
	2 vaccine doses	0.80 (0.66, 0.96)	0.74 (0.58, 1.18)
	3 vaccine doses	0.62 (0.57, 0.66)	0.58 (0.37, 0.70)
	4 vaccine doses	0.53 (0.47, 0.72)	0.36 (0.20, 0.59)
	No documented prior infection	ref.	ref.
	Any documented prior infection	0.78 (0.68, 0.92)	0.73 (0.46, 1.30)
BA.2 cases			
	0 vaccine doses	ref.	ref.
	2 vaccine doses	0.80 (0.64, 0.98)	0.87 (0.67, 1.44)
	3 vaccine doses	0.74 (0.67, 0.79)	0.65 (0.39, 0.81)
	4 vaccine doses	0.60 (0.52, 0.86)	0.51 (0.25, 0.90)
	No documented prior infection	ref.	ref.
	Any documented prior infection	0.70 (0.57, 0.88)	0.77 (0.44, 1.57)

CI: Confidence interval. Estimates indicate the adjusted hazard ratios (aHR) of each outcome, comparing cases with BA.4/BA.5 infection to those with BA.2 infection, estimated via Cox proportional hazards models including strata for cases' week of diagnosis and all covariates listed in **Table 1**.
[2]Previous infection defined by any positive test result or diagnosis ≥90 days prior to the date of the current test. We omit estimates among recipients of single vaccine doses due to sparse sample sizes (*N*=1,121 BA.4/BA.5 cases and 1,503 BA.2 cases).

Table S8: Prevalence of prior documented infection among newly-diagnosed cases, as compared to all KSPC members.

**Month**	**Prevalence of prior infection**[1]	
	All KPSC members	*Newly diagnosed cases*
January, 2022	8.6%	1.1%
February, 2022	8.9%	1.6%
March, 2022	9.1%	1.6%
April, 2022	10.9%	2.6%
May, 2022	15.7%	2.8%
June, 2022	16.0%	3.8%
July, 2022	16.0%	5.7%

[1]Defined as a prior positive SARS-CoV-2 result in any clinical setting in the preceding ≥90 days; for reports among all KPSC members, counts of members with a prior infection are defined as ≥90 days before the first day of each month indicated.

Table S9: Clinical outcomes among cases with BA.2 and BA.4/BA.5 lineage SARS-CoV-2 infection with documented history of SARS-CoV-2 infection.

Clinical outcome	Population	Events, *n* (%)[1]	Rate per 100,000 person-days	HR (95% CI)	aHR (95% CI)
Emergency department presentation—15 days					
	BA.2 (*S gene detected*)	26 (1.8)	122.9	ref.	ref.
	BA.4/BA.5 (*S gene target failure*)	54 (1.7)	118.8	0.80 (0.44, 1.48)	0.81 (0.43, 1.52)
Emergency department presentation—30 days					
	BA.2 (*S gene detected*)	49 (3.4)	117.1	ref.	ref.
	BA.4/BA.5 (*S gene target failure*)	89 (2.8)	100.0	0.85 (0.54, 1.35)	0.87 (0.54, 1.41)
Hospital admission—15 days					
	BA.2 (*S gene detected*)	4 (0.3)	18.1	ref.	– –
	BA.4/BA.5 (*S gene target failure*)	6 (0.2)	13.0	0.80 (0.13, 5.06)	– –
Hospital admission—30 days					
	BA.2 (*S gene detected*)	7 (0.5)	16.2	ref.	ref.
	BA.4/BA.5 (*S gene target failure*)	11 (0.4)	12.2	1.30 (0.33, 5.09)	1.45 (0.29, 7.14)
ARI-associated hospital admission—30 days					
	BA.2 (*S gene detected*)	4 (0.3)	9.3	ref.	– –
	BA.4/BA.5 (*S gene target failure*)	3 (0.1)	3.3	0.49 (0.06, 4.39)	– –

CI: Confidence interval. Estimates indicate the hazard ratio (HR) adjusted hazard ratio (aHR) of each outcome, comparing cases with BA.4/BA.5 infection to those with BA.2 infection, estimated via Cox proportional hazards models including strata for cases' calendar week (or weekend) of diagnosis and all covariates listed in **Table 1** and **Table 2**. Analyses are subset to cases with documented SARS-CoV-2 infection based on a positive molecular test result ≥90 days before the date of the current test (3,139 BA.4/BA.5 cases and 1,458 BA.2 cases). We omit analyses of endpoints of ICU admission, mechanical ventilation, and death due to sparse counts.

Table S10: Estimation of excess risk of hospital admission attributable to SARS-CoV-2 infection.

Source	Risk of COVID-19 associated hospital admission[1]	Rate of hospital admission due to COVID-19[1]		Rate of hospital admission due to Omicron variant[2]	Incidence rate ratio of hospital admission during SARS-CoV-2 infection[3]	
	Per infection, cumulative	*Per day, per 100*	*Annualized, per 100*	*Annualized, per 100*	*Pre-Omicron*	*Omicron variant era*
Mahajan et al., 2021 (Connecticut)[4]	6.9%	0.41	148.1	43.3	20.0	5.9
Barber et al. 2022 (US-wide)[5]	3.5%	0.21	75.1	22.0	10.2	3.0
Barber et al., 2022 (California)[5]	3.3%	0.19	70.9	20.7	9.6	2.8
Goss et al., 2022 (Missouri)[6]	4.9%	0.29	105.2	30.8	14.2	4.2

[1]Data from included studies measure the infection-to-hospitalization ratio by dividing COVID-19 associated hospitalizations (defined as those associated with a positive diagnostic test) by cumulative infections, typically measured based on serological surveillance. We obtain the daily risk of hospital admission by dividing cumulative risk of COVID-19 associated hospital admission from each study by the mean 17-day duration of molecular test positivity for SARS-CoV-2, conveying the period over which infection-associated hospital admissions would be expected to be captured (He et al., *Nature Med* 2020; doi: 10.1038/s41591-020-0869-5). We multiply the resulting daily risk of hospital admission by 365 to obtain annualized rates for comparison to pre-pandemic data on rates of all-cause hospital admissions.

[2]We accounted for the reduced severity of Omicron variant infections and the protective effects of prevalent naturally-acquired and vaccine-derived immunity by multiplying annualized rates of hospital admission due to COVID-19 by 0.29, representing the relative risk of hospital admission per infection in Spring, 2022 as compared to May, 2020 (Eales et al., *medRxiv* 2022; doi:10.1101/2022.10.12.22281016).

[3]We obtained incidence rate ratios by dividing the estimated annualized risk of hospitalization during SARS-CoV-2 infection (with and without the Omicron variant correction described above) by the pre-pandemic rate of 7.6 hospital admissions per 100 person-years in the US (National Health Interview Survey data, available from: https://www.cdc.gov/nchs/hus/topics/hospitalization.htm).

[4]Mahajan et al., *Am J Med* 2021; doi:10.1016/j.amjmed.2021.01.020.

[5]Barber et al., *Lancet* 2022; doi:10.1016/S0140-6736(22)00484-6.

[6]Goss et al., *Ann Epidemiol* 2022; doi: 10.1016/j.annepidem.2022.03.002.

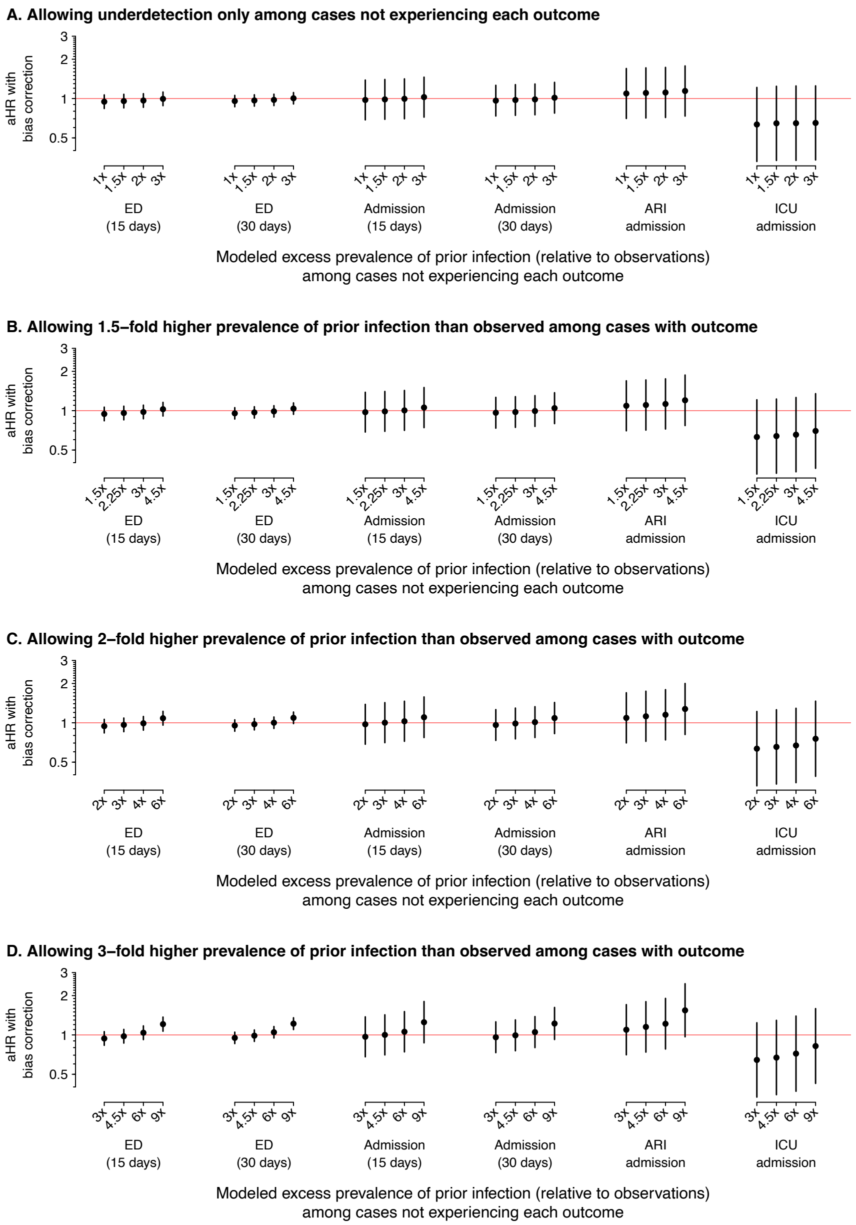

Figure S1: We present estimates of the bias-corrected adjusted hazards ratio (aHR), together with 95% confidence intervals (CIs; vertical lines), under various scenarios with respect to the proportion of prior infections observed among individuals who experienced each outcome (ρ^{-1}, for $\rho \in \{1, 1.5, 2, 3\}$) and among individuals who did not experience each outcome (α^{-1}, for $\alpha \in \{1, 1.5, 2, 3\}$). Individuals' baseline propensity for having experienced prior infection is estimated by logistic regression, thus accounting for risk factors including vaccination and the individual's infection with either the BA.4/BA.5 or BA.2 lineages. Panels **a**, **b**, **c**, **d** correspond to scenarios with $\rho = 1, 1.5, 2, 3$, respectively, among individuals who experienced each outcome, while estimates under differing values of α are presented side-by-side within each panel, for each endpoint. Row labels denote the product $\alpha \times \rho$, indicating the modeled ratio of true-to-observed infections among individuals who did not experience each clinical endpoint of interest. Statistically-significant bias-corrected aHR estimates (signified by 95% CIs that exclude the null hypothesis of no difference in risk of each outcome for cases with BA.4/BA.5 or BA.2 lineage infections) emerge only under the scenario of $\rho = 3$ and $\alpha = 3$, for 15- and 30-day risk of ED presentation. Following bias correction, we estimate that the aHR or ED presentation comparing BA.4/BA.5 to BA.2 cases is 1.21 (95% CI: 1.07-1.37) over 15 days and 1.22 (1.10-1.35) over 30 days under this scenario. Data encompass outcomes among 106,532 SARS-CoV-2 cases (49,976 with BA.2 infections and 59,556 with BA.4/BA.5 infections). Lines indicate 95% confidence intervals around medians (center points).
