# Supplementary material for: Association of SARS-CoV-2 BA.4/BA.5 Omicron lineages with immune escape and clinical outcome
Source: Nat Commun. 2023 Mar 14;14:1407. doi: 10.1038/s41467-023-37051-5 (PMC10012300; doi:10.1038/s41467-023-37051-5)
Supplement: Supplementary file 3 — Reporting Summary [file 41467_2023_37051_MOESM3_ESM.pdf]

nature portfolio

Corresponding author(s): Joseph Lewnard

Last updated by author(s): Feb 15, 2023

# Reporting Summary

Nature Portfolio wishes to improve the reproducibility of the work that we publish. This form provides structure for consistency and transparency in reporting. For further information on Nature Portfolio policies, see our Editorial Policies and the Editorial Policy Checklist.

## Statistics

For all statistical analyses, confirm that the following items are present in the figure legend, table legend, main text, or Methods section.

| n/a | Confirmed | |
|---|---|---|
| ☐ | ☒ | The exact sample size (*n*) for each experimental group/condition, given as a discrete number and unit of measurement |
| ☐ | ☒ | A statement on whether measurements were taken from distinct samples or whether the same sample was measured repeatedly |
| ☒ | ☐ | The statistical test(s) used AND whether they are one- or two-sided<br>*Only common tests should be described solely by name; describe more complex techniques in the Methods section.* |
| ☐ | ☒ | A description of all covariates tested |
| ☐ | ☒ | A description of any assumptions or corrections, such as tests of normality and adjustment for multiple comparisons |
| ☐ | ☒ | A full description of the statistical parameters including central tendency (e.g. means) or other basic estimates (e.g. regression coefficient) AND variation (e.g. standard deviation) or associated estimates of uncertainty (e.g. confidence intervals) |
| ☒ | ☐ | For null hypothesis testing, the test statistic (e.g. *F*, *t*, *r*) with confidence intervals, effect sizes, degrees of freedom and *P* value noted<br>*Give P values as exact values whenever suitable.* |
| ☒ | ☐ | For Bayesian analysis, information on the choice of priors and Markov chain Monte Carlo settings |
| ☒ | ☐ | For hierarchical and complex designs, identification of the appropriate level for tests and full reporting of outcomes |
| ☒ | ☐ | Estimates of effect sizes (e.g. Cohen's *d*, Pearson's *r*), indicating how they were calculated |

*Our web collection on statistics for biologists contains articles on many of the points above.*

## Software and code

Policy information about availability of computer code

| | |
|---|---|
| Data collection | No software was used for data collection. |
| Data analysis | We conducted all analyses using R (version 4.0.3; R Foundation for Statistical Computing, Vienna, Austria). We used the survival package (version 3.5-3) for time-to-event analyses, and the Amelia II package (version 1.8.1) for multiple imputation. Analysis code is available from github.com/joelewnard/ba4ba5severity. |

For manuscripts utilizing custom algorithms or software that are central to the research but not yet described in published literature, software must be made available to editors and reviewers. We strongly encourage code deposition in a community repository (e.g. GitHub). See the Nature Portfolio guidelines for submitting code & software for further information.

## Data

Policy information about availability of data

All manuscripts must include a data availability statement. This statement should provide the following information, where applicable:

- Accession codes, unique identifiers, or web links for publicly available datasets
- A description of any restrictions on data availability
- For clinical datasets or third party data, please ensure that the statement adheres to our policy

Individual-level data reported in this study are not publicly shared. Upon request and subject to review by the KPSC Institutional Review Board, KPSC may provide

the de-identified aggregate-level data that support the findings of this study. De-identified data may be shared upon approval of an analysis proposal and a signed data access agreement. The corresponding authors (JAL, SYT) will respond to requests for data access within 14 days of receipt.

## Human research participants

Policy information about studies involving human research participants and Sex and Gender in Research.

| | |
|---|---|
| Reporting on sex and gender | We indicate that sex is measured as a biological variable and report on biological sex referring to individuals as males and females. As indicated in Table 1, the sample includes 25,905 females and 21,071 males among BA.2 cases, and 32,302 females and 27,254 males among BA.4/BA.5 cases. |
| Population characteristics | Age, sex, race, socioeconomic status (measured at the community level), smoking behavior, body mass index, comorbidities, and healthcare utilization variables within the population are tabulated in Table 1 for BA.4/BA.5 and BA.2 cases. |
| Recruitment | This analysis included all individuals who are members of Kaiser Permanente Southern California health plans (who had been continuously enrolled for ≥1 year at the time of the study, to support evaluation of comorbidities and healthcare utilization) who received a positive molecular diagnostic test for SARS-CoV-2 during the study period. Individuals in this insured cohort may have better healthcare access than those without commercial insurance. Additionally, the decision to seek outpatient SARS-CoV-2 testing may differ with other parameters of healthcare utilization within the population enrolled in KPSC health plans. Individuals who sought testing in clinical settings during this time, when home antigen testing was widely available, may not be representative of all individuals who acquired SARS-CoV-2 infection, leading to selection bias. However, we are unaware of reasons that such bias would be differential among cases infected with BA.2 and BA.4/BA.5 |
| Ethics oversight | The study protocol was approved by the KPSC Institutional Review Board. |

Note that full information on the approval of the study protocol must also be provided in the manuscript.

# Field-specific reporting

Please select the one below that is the best fit for your research. If you are not sure, read the appropriate sections before making your selection.

☒ Life sciences ☐ Behavioural & social sciences ☐ Ecological, evolutionary & environmental sciences

For a reference copy of the document with all sections, see nature.com/documents/nr-reporting-summary-flat.pdf

# Life sciences study design

All studies must disclose on these points even when the disclosure is negative.

| | |
|---|---|
| Sample size | For this observational study, the sample size was not pre-determined as interventions were not administered by researchers; all patients meeting eligibility criteria with COVID-19 diagnoses during the study period were included in analyses. Within this large sample population (46,976 BA.2 cases and 59,556 BA.4/BA.5 cases), among ~4.7 million members of Kaiser Permanente Southern California health plans, sufficient power was available for estimation of even small effect sizes. |
| Data exclusions | We restricted our analytic sample to individuals who first tested positive in an outpatient setting to select on healthcare-seeking behavior within the study population, thus maximizing internal validity when comparing outcomes among BA.4/BA.5 and BA.2 cases. Analyses included individuals' first positive test result (defined as the index test) during 2022 to support interpretation of S gene target failure and infecting lineage. In total, 106,532 SARS-CoV-2 cases out of 148,105 diagnosed as outpatients at KPSC during the study period met eligibility criteria and were included in analyses. We excluded 18,799 patients without ≥1 year of continuous enrollment before their positive test (enabling determination of risk factors and characterization of healthcare seeking behavior), and 22,774 whose tests were not processed using the ThermoFisher TaqPath COVID-19 Combo Kit (which enabled lineage determination based on S gene target failure). |
| Replication | We undertook sensitivity analyses exploring two alternative endpoints (1: ED presentation within 15 days; and 2: hospital admission within 15 days), and compared primary results to those obtained within the stratum of individuals who had documented history of SARS-CoV-2 infection to ensure outcomes were not driven by misclassification of prior infection status (which could not explain results within this stratum). Each of these analyses yielded results consistent with those of the primary analyses. Last, we undertook risk-of-bias analyses exploring how differential misclassification of prior infection status could impact estimates of associations of clinical outcomes with infecting lineage. These analyses consisted of 16 alternative scenarios with respect to the relative likelihood of prior infection among all cases (1, 1.5, 2 and 3x higher than observed) and the enhancement in likelihood of prior infection among cases who did not experience severe outcomes (1, 1.5, 2, and 3x higher than observed), and evaluated impacts on all study endpoints (ED presentation within 15 or 30 days, hospital admission within 15 or 30 days, symptomatic hospital admission within 30 days, and ICU admission). These analyses did not identify results in conflict with the conclusions of the primary analysis.<br><br>As our analysis included multiple imputation of missing observations, all analyses were repeated across 10 pseudo-datasets with missing observations drawn from their conditional distributions with respect to other observed variables. Reported results are pooled across these analyses. |
| Randomization | Analyses controlled for the following characteristics for each case via covariate adjustment: age (defined in 10-year age bands), sex, race/ethnicity (white, black, Hispanic of any race, Asian, Pacific Islander, and other/mixed/unknown race), neighborhood deprivation index, |

measured at the Census block level; smoking status (current, former, or never smoker); body mass index (BMI; underweight, normal weight, overweight, obese, and morbidly obese); Charlson comorbidity index (0, 1-2, 3-5, and ≥6); prior-year emergency department visits and inpatient admissions (each defined as 0, 1, 2, or ≥3 events); prior- year outpatient visits (0-4, 5-9, 10-14, 15-19, 20-29, or ≥30 events); documented prior SARS-CoV-2 infection; and history of COVID-19 vaccination (receipt of 0, 1, 2, 3, or ≥4 doses, and time from receipt of each dose to each case's testing date), and receipt of Paxlovid ≤14 days after the initial outpatient diagnosis date. Regression models defined strata for the week of SARS-CoV-2 testing to further control for changes in testing practices.

Blinding

While the study was not strictly blinded, determinations of S gene target failure (proxy for BA.2 or BA.4/BA.5 infection) were not included in patients' clinical record; thus, clinical personnel were unaware of whether patients' infecting lineage. As infecting lineage does not inform clinical management, such data were not considered relevant to clinical care provision. Furthermore, analyses linking S gene target failure results to patients' clinical data were undertaken retrospectively, after clinical follow-up of patients had already been completed. Data analysts were not blinded to cases' status of S gene target failure or S gene detection. Blinding of data analysts was not considered feasible due to the need for direct correspondence with clinical laboratory personnel, including the data management team.

# Reporting for specific materials, systems and methods

We require information from authors about some types of materials, experimental systems and methods used in many studies. Here, indicate whether each material, system or method listed is relevant to your study. If you are not sure if a list item applies to your research, read the appropriate section before selecting a response.

## Materials & experimental systems

| n/a | Involved in the study | |
|---|---|---|
| ☒ | ☐ | Antibodies |
| ☒ | ☐ | Eukaryotic cell lines |
| ☒ | ☐ | Palaeontology and archaeology |
| ☒ | ☐ | Animals and other organisms |
| ☐ | ☒ | Clinical data |
| ☒ | ☐ | Dual use research of concern |

## Methods

| n/a | Involved in the study | |
|---|---|---|
| ☒ | ☐ | ChIP-seq |
| ☒ | ☐ | Flow cytometry |
| ☒ | ☐ | MRI-based neuroimaging |

## Clinical data

Policy information about clinical studies

All manuscripts should comply with the ICMJE guidelines for publication of clinical research and a completed CONSORT checklist must be included with all submissions.

Clinical trial registration

Not applicable

Study protocol

A prespecified protocol was not generated for this observational study, which was undertaken in real time with emergence of the BA.4/BA.5 lineages.

Data collection

For this observational study, data were as part of routine care provision as part of patients' electronic health records. In-network care delivery data encompassing diagnoses (and accompanying clinical notes), immunizations, laboratory tests administered and test results, and prescriptions are captured in near-real time via patient EHRs, while out-of-network care is captured through insurance claim reimbursements. Delivery of COVID-19 vaccine doses by other providers was identified via linkage to California Immunization Registry data and other health systems using the Epic EHR system. Online portals provide an automated platform for individuals to upload or notify providers of positive at-home test results or test results received from other providers.

Outcomes

Outcomes of interest to our analyses included: (1) any ED presentation; (2) any inpatient admission; (3) ARI- associated inpatient admission, at which physicians recorded ≥1 of the ARI diagnostic codes indicated in Table S1; (4) ICU admission; (5) mechanical ventilation; and (6) death. We limited follow-up time for ED presentation and hospital admission to 30 days following the initial positive outpatient test; we included follow-up time through 60 days from the initial positive outpatient test for endpoints of ICU admission, mechanical ventilation, and death, owing to the longer course of disease expected to precede such outcomes. We censored observations at study end date or at disenrollment for cases who had not experienced each outcome. As cases diagnosed in outpatient settings were enrolled in a home- based monitoring program with standardized criteria for ED referral and inpatient admission, we expected severity of illness associated with each endpoint to be internally comparable within the study cohort. Last, to facilitate our ability to measure intrinsic associations of infecting lineage with risk of progression to severe outcomes, we censored observations at dates of Paxlovid dispense for individuals who received this treatment (5.5% of patients analyzed [n=5,833]).
